# Uptake of fluorescent d- and l-glucose analogues, 2-NBDG and 2-NBDLG, into human osteosarcoma U2OS cells in a phloretin-inhibitable manner

**DOI:** 10.1007/s13577-020-00483-y

**Published:** 2021-01-17

**Authors:** Tetsuya Ogawa, Ayako Sasaki, Koki Ono, Shusa Ohshika, Yasuyuki Ishibashi, Katsuya Yamada

**Affiliations:** 1 Department of Orthopaedic Surgery, Hirosaki University Graduate School of Medicine, 5 Zaifu-cho, Hirosaki, Aomori, 036-8562 Japan; 2 Department of Physiology, Hirosaki University Graduate School of Medicine, 5 Zaifu-cho, Hirosaki, Aomori, 036-8562 Japan

**Keywords:** l-glucose, Tumor, Sarcoma, Imaging, Transport

## Abstract

Mammalian cells take in d-glucose as an essential fuel as well as a carbon source. In contrast, l-glucose, the mirror image isomer of d-glucose, has been considered merely as a non-transportable/non-metabolizable control for d-glucose. We have shown that 2-[*N*-(7-Nitrobenz-2-oxa-1,3-diazol-4-yl)amino]-2-deoxy-D-glucose (2-NBDG), a d-glucose analogue combining a fluorophore NBD at the C-2 position, is useful as a tracer for monitoring d-glucose uptake through glucose transporters (GLUTs) into mammalian cells. To more precisely evaluate the stereoselectivity of 2-NBDG uptake, we developed an l-glucose analogue 2-NBDLG, the mirror-image isomer of 2-NBDG. Interestingly, 2-NBDLG was taken up into mouse insulinoma MIN6 cells showing nuclear heterogeneity, a cytological feature of malignancy, while remaining MIN6 cells only exhibited a trace amount of 2-NBDLG uptake. The 2-NBDLG uptake into MIN6 cells was abolished by phloretin, but persisted under blockade of major mammalian glucose transporters. Unfortunately, however, no such uptake could be detected in other tumor cell lines. Here we demonstrate that human osteosarcoma U2OS cells take in 2-NBDLG in a phloretin-inhibitable manner. The uptake of 2-NBDG, and not that of 2-NBDLG, into U2OS cells was significantly inhibited by cytochalasin B, a potent GLUT inhibitor. Phloretin, but neither phlorizin, an inhibitor of sodium-glucose cotransporter (SGLT), nor a large amount of d/l-glucose, blocked the 2-NBDLG uptake. These results suggest that a phloretin-inhibitable, non-GLUT/non-SGLT, possibly non-transporter-mediated yet unidentified mechanism participates in the uptake of the fluorescent l-glucose analogue in two very different tumor cells, the mouse insulinoma and the human osteosarcoma cells.

**Supplementary Information:**

The online version contains supplementary material available at 10.1007/s13577-020-00483-y.

## Introduction

d-glucose, the minimum unit of starch, is one of the most fundamental nutrients for living things. Mammalian cells take d-glucose into the cytosol through facilitated glucose transporters (GLUTs) or sodium-glucose cotransporters (SGLTs) [1, 2]. On the contrary, l-glucose could not be taken in nor metabolized by cells except some soil bacteria [3]. As such, l-glucose has been used only as a negative control substrate for d-glucose [4].

For investigating d-glucose uptake into cells, radio-labeled analogues of d-glucose, such as [^18^F]2-fluoro-2-deoxy-d-glucose (FDG) which attaches fluorine at the C-2 position of the glucose, have been used widely [5]. FDG is particularly effective as a tracer for imaging cancer in combination with the positron emission tomography (PET), although early diagnosis of cancer with the FDG-PET is difficult due to its low spatial resolution [5]. In addition, the radiation exposure has made its use limited [6].

**Fig. 1:**
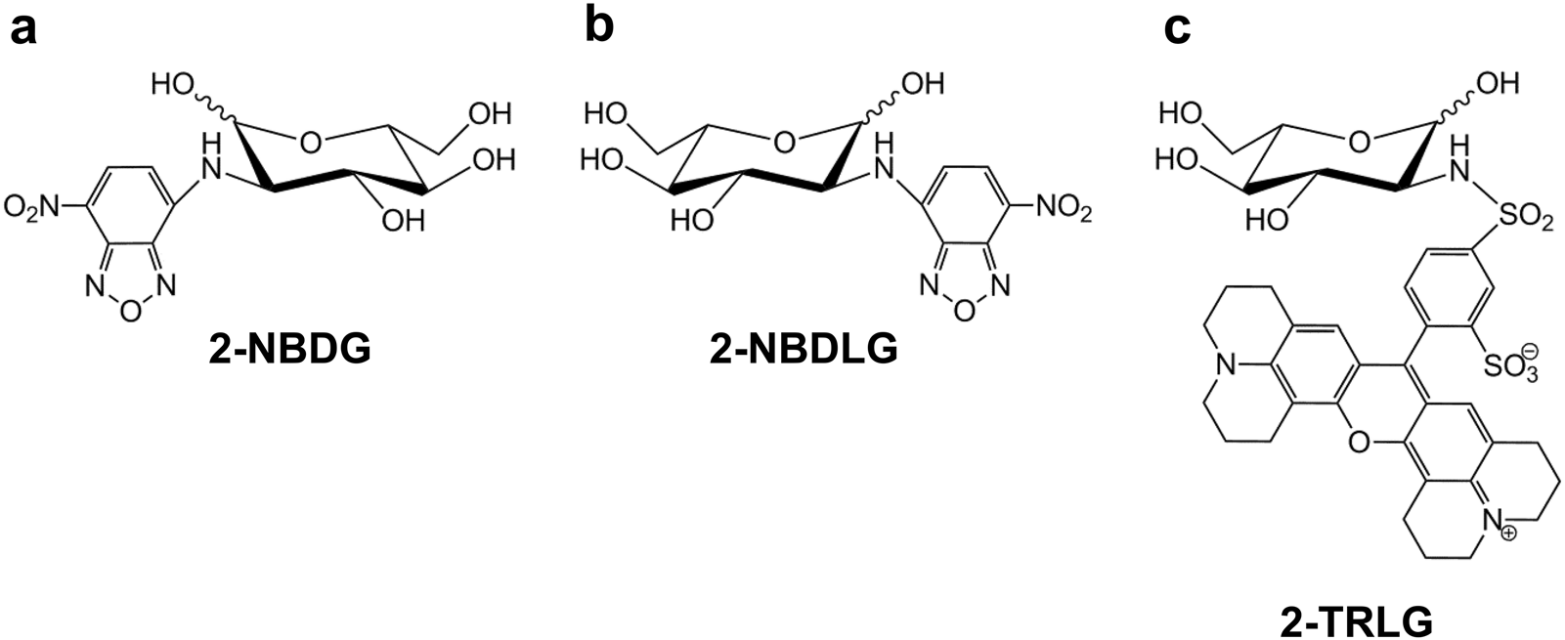
Chemical structures of 2-NBDG (**a**), 2-NBDLG (**b**) and 2-TRLG (**c**)

To overcome such difficulties, use of fluorescently labeled analogues of glucose is another possibility [7–10]. We have demonstrated that d-glucose analogues such as 2-NBDG (Fig. 1a) bearing a small green fluorophore NBD, or CDG bearing a blue fluorophore coumarin, at the C-2 position enter mammalian cells through GLUTs, and are effective tracers for monitoring d-glucose uptake in single living cells [7, 11, 12]. Several investigators have also reported that 2-NBDG uptake occurred also through SGLTs [13, 14]. This is a strong point of 2-NBDG as a tracer for monitoring d-glucose uptake considering the fact that FDG does not permeate SGLTs [15].

2-NBDG has been proposed to visualize tumor cells as well [16]. However, there may be a number of ways that cells use to take in glucose [4]. Thus, to more precisely understand this issue, we developed a fluorescently labeled l-glucose analogue 2-[*N*-(7-Nitrobenz-2-oxa-1,3-diazol-4-yl) amino]-2-deoxy-L-glucose (2-NBDLG, Fig. 1b), the mirror image isomer of 2-NBDG, as a negative control [17].

Beyond our expectations, 2-NBDLG was specifically taken up into mouse insulinoma MIN6 cells when they exhibited nuclear heterogeneity, a clinical sign of malignancy in cytological diagnosis [18]. Pharmacological experiments further revealed that both of the inhibitors against GLUTs and against SGLTs failed to suppress the 2-NBDLG uptake into MIN6 cells [18]. Eventually, we found that the uptake of 2-NBDLG was abolished by phloretin [18], an aglycone of natural polyphenol phlorizin and a broad-spectrum inhibitor against membrane transport including GLUTs/water channels [19, 20], implying that 2-NBDLG enters the tumor cells through non-GLUT/non-SGLT pathways [4]. Since then, we had been unable to find out other tumor cell lines showing such specific uptake of 2-NBDLG.

We show in the present study that human osteosarcoma U2OS cells take up 2-NBDLG into cells with pharmacological properties very similar to those we reported in MIN6 cells. Osteosarcoma is an aggressive, primary bone sarcoma that occurs mostly in teenagers and young adults [21, 22]. When it has metastasized or relapsed, the 5-year survival rate is reported to be about 20% [22]. Its rarity, only one to three cases annually per million in incidence worldwide [22], histological/genomic complexity, and diverse clinical behaviors are among major factors causing difficulties in diagnosis and treatment of osteosarcoma [23–25]. In addition, no imaging modality has been available for reliably discriminating malignant bone/soft tissue tumors from benign ones at the single cell study [26]. 2-NBDLG is one of the candidate tracers for visualizing single living tumor cells expressing malignant phenotypes [4]. To our best knowledge, this is the first study to characterize a human tumor cell line that is capable of specifically taking up the fluorescent analogue of l-glucose into cells in a pharmacologically defined manner.

## Methods

### Confocal microscopic imaging

#### Culture

Osteosarcoma U2OS (HTB-96, ATCC) cells were cultured using RPMI 1640 medium (11875-093, Gibco) containing 10% Fetal Bovine Serum (26140-079, Gibco) and 1% Penicillin–Streptomycin (15140-122, Gibco). Cells in passages from 32–36th were used in the experiment. 10 μl of U2OS cell suspension (5000 cells/ml) was seeded on small glass coverslips (2.5 mm times 7.0 mm, No. 0, Matsunami Glass Ind., Ltd.). After leaving cells for 60 min in a CO_2_ incubator at 37 ºC for ensuring stable attachment, 500 μl of the culture medium was carefully added to each well. Culture medium was half exchanged every 3 days.

#### Measurement

Confocal microscopy was conducted to visualize difference in the uptake of the fluorescent d- and l-glucose tracers in U2OS cells. Cells used for the measurement were cultured for 7 days in vitro (DIV) when an adequate number of cells maintaining healthy condition were obtained for each coverslip at the concentration seeded. The tracer administration and image acquisition were conducted by modifying a method reported previously (see Online Resource 4 for details) [7, 27].

### Pharmacological evaluation of the tracer uptake by a fluorescence microplate reader

#### Culture and measurement

Details in the culturing and experimental procedures were similar to those reported previously (see Online Resource 4) [18].

#### Reagents

2-NBDLG (23003-v, Peptide Institute), 2-NBDG (23002-v, Peptide Institute), 2-TRLG (Peptide Institute), phloretin (P7912, Sigma), cytochalasin B (C6762, Sigma), phlorizin (P3449, Sigma) and 4′,6-diamidino-2-phenylindole (DAPI, 049-18801, Wako) were prepared as described previously [18]. Cytochalasin B was used to examine an involvement of GLUTs in the uptake of glucose tracers, since it acts as a specific antagonist of GLUTs when applied briefly in a low dose [11]. Phlorizin was used to inhibit SGLTs [2]. In all experiments, 100 μM of carbenoxolone (C4790, Sigma) was routinely added to exclude non-specific permeation of the fluorescent tracers through gap junctions/hemichannels [28]. d-glucose and l-glucose were purchased from Fujifilm Wako Pure Chemical (049-31165) and Tokyo Chemical Industry (921-60-8), respectively.

### Statistics

Unpaired *t* test or Bonferroni/Dunn test were used for statistical analyses. All the values represent mean fluorescence intensity, and are expressed as mean ± SD.

## Results

### Evaluation of 2-NBDG and 2-NBDLG uptake into U2OS cells by confocal microscopy

**Fig. 2:**
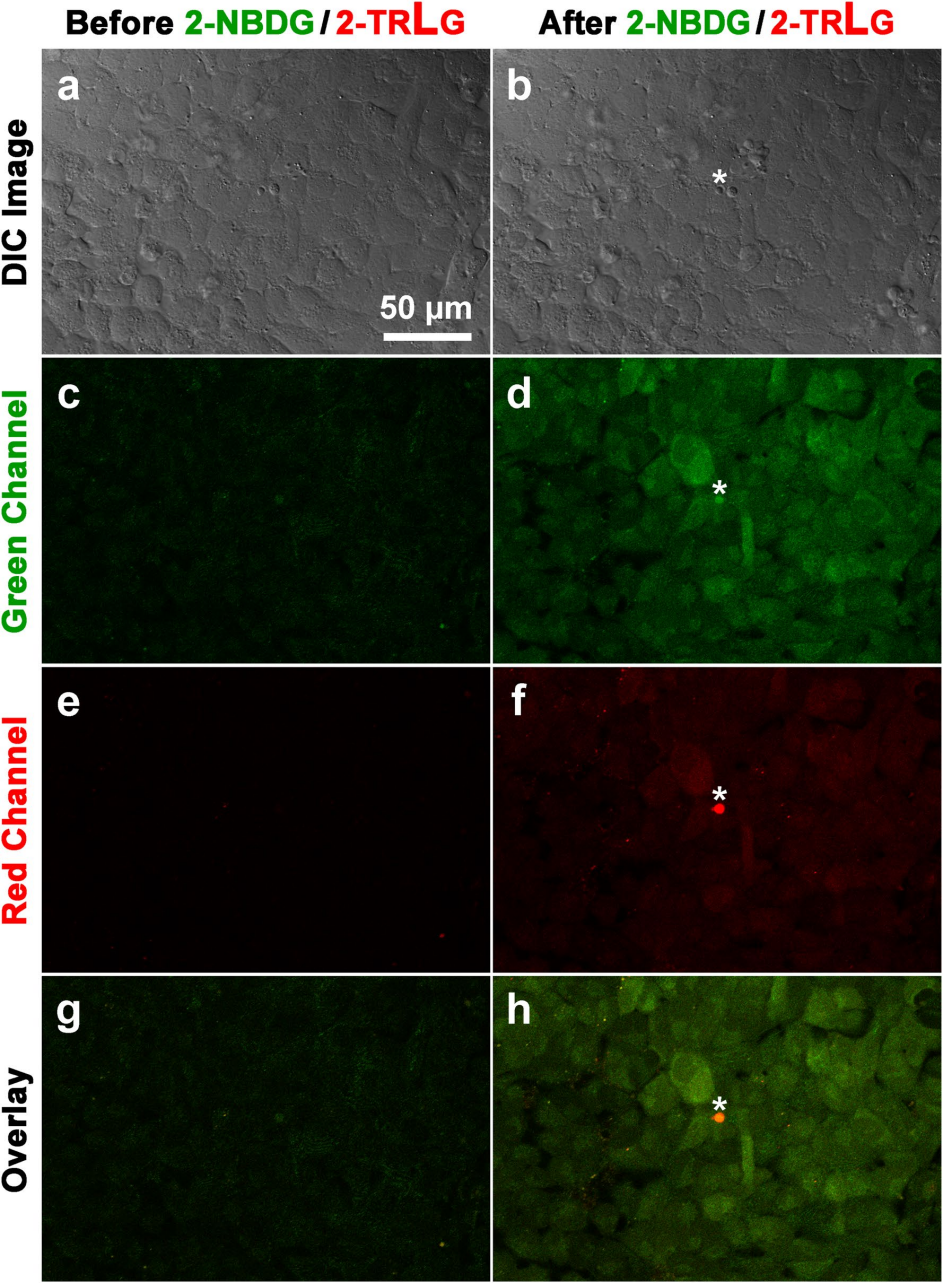
Representative images of U2OS cells subjected to 2-NBDG and 2-TRLG for 5 min followed by washout on the stage of confocal microscope at 7 DIV. **a**, **b** Differential interference contrast (DIC) images taken before administration (**a**) and 6 min after washout (**b**) of KRB solution containing a mixture of 200 μM 2-NBDG, which emits a large fluorescence in 500–580 nm (green channel) and a small fluorescence in 580–740 nm (red channel), and 20 μM 2-TRLG, which emits fluorescence solely in 580–740 nm (red channel) and was added for detecting non-specific entry of the fluorescent tracers due to a loss of membrane integrity. **c, d** Similar to (**a**,** b**), but fluorescence images taken in the green channel. **e**, **f** Similar to (**c**, **d**), but that in the red channel. **g** Superimposed images of (**c**) and (**e**). **h** Similar to (**g**), but for (**d**) and (**f**). An asterisk denotes small debris. The bar is common to all panels. Note heterogeneity in the fluorescence intensity among cells

To visually inspect uptake of a d-glucose tracer 2-NBDG in U2OS osteosarcoma cells, confocal microscopic imaging was conducted. On the stage of the microscope, U2OS cells were transiently superfused for 5 min with KRB solution containing 200 μM of green fluorescence-emitting 2-NBDG together with 20 μM of red fluorescence-emitting 2-TRLG at 37 ºC. 2-TRLG, which is a membrane impermeable l-glucose analogue [27], was used here to identify an occurrence of non-specific entry of the green tracer 2-NBDG (or 2-NBDLG) due to a loss of membrane integrity [18]. A comparison of the green fluorescence intensity of U2OS cells before and after administration of the 2-NBDG/2-TRLG mixture demonstrated that the fluorescence remarkably increased compared to that detected before administration (Fig. 2a–d). The average fluorescence intensity of cells in the green channel was evaluated for seven pre-determined areas (14–24 ROIs for each area), demonstrating that the intensity in the green channel increased significantly after administration of the mixture (584.2 ± 146.1 arbitrary unit, hereinafter abbreviated as AU) from the value before (231.9 ± 29.1 AU, with the number of ROIs = 134, *p* < 0.0001, Fig. 4a).

Along with the increase in the fluorescence of the green channel, a small increase was also detected in the fluorescence of the red channel (Fig. 2e, f). However, except for small debris emitting strong red fluorescence (asterisk), the relative pattern of the fluorescence in the red channel (Fig. 2f) was proportional to that in the green channel (Fig. 2d), indicating that the red fluorescence of cells, except for the debris, reflected the fluorescence component of 2-NBDG in the wavelength longer than 580 nm [27]. Interpreted alternatively, the increase in the 2-NBDG fluorescence in U2OS cells was not due to non-specific entry [18] of the tracer (Fig. 2g, h).

**Fig. 3:**
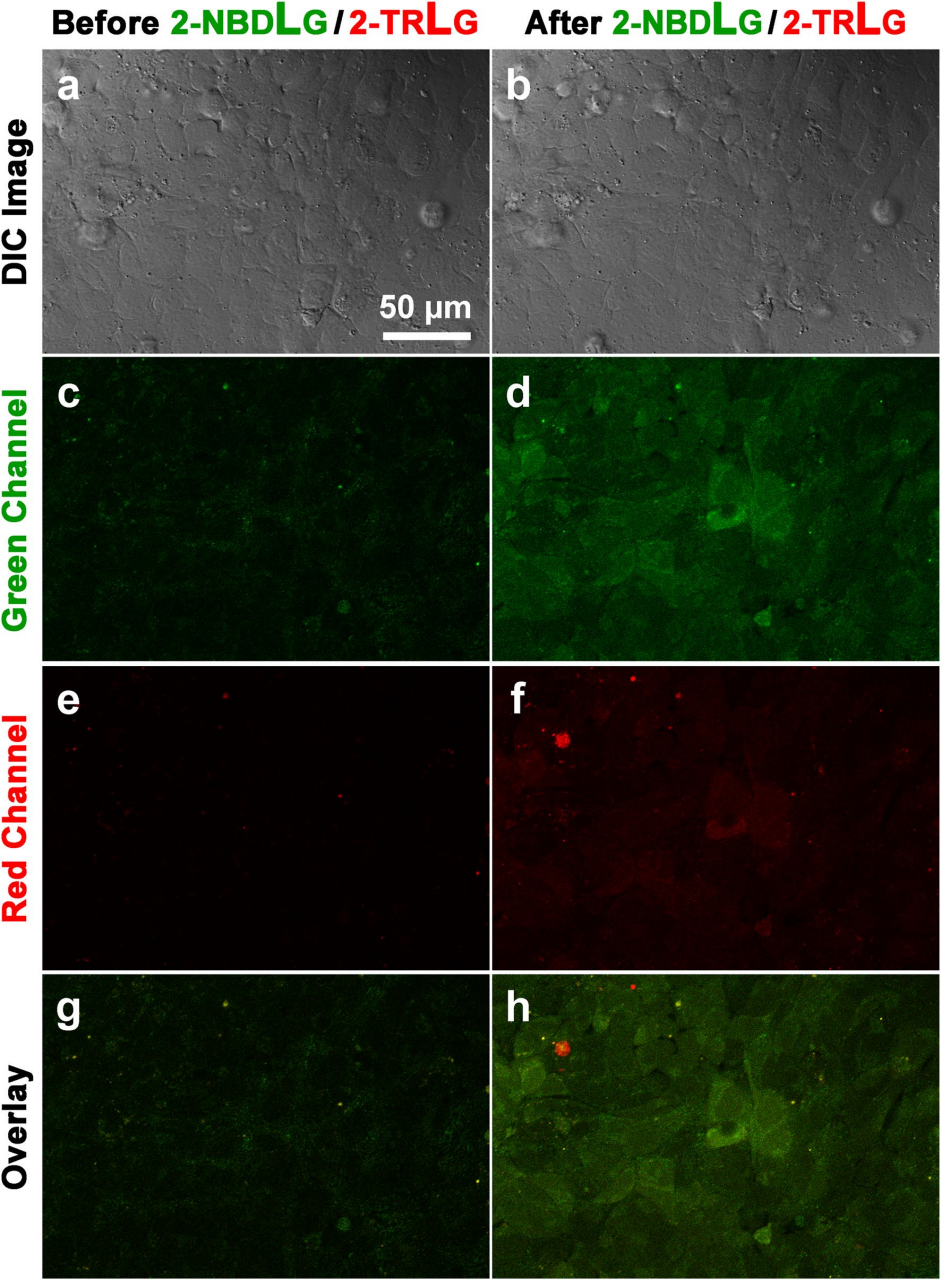
Similar to Fig. 2, but for administration of KRB solution containing 200 μM of 2-NBDLG and 20 μM of 2-TRLG

**Fig. 4:**
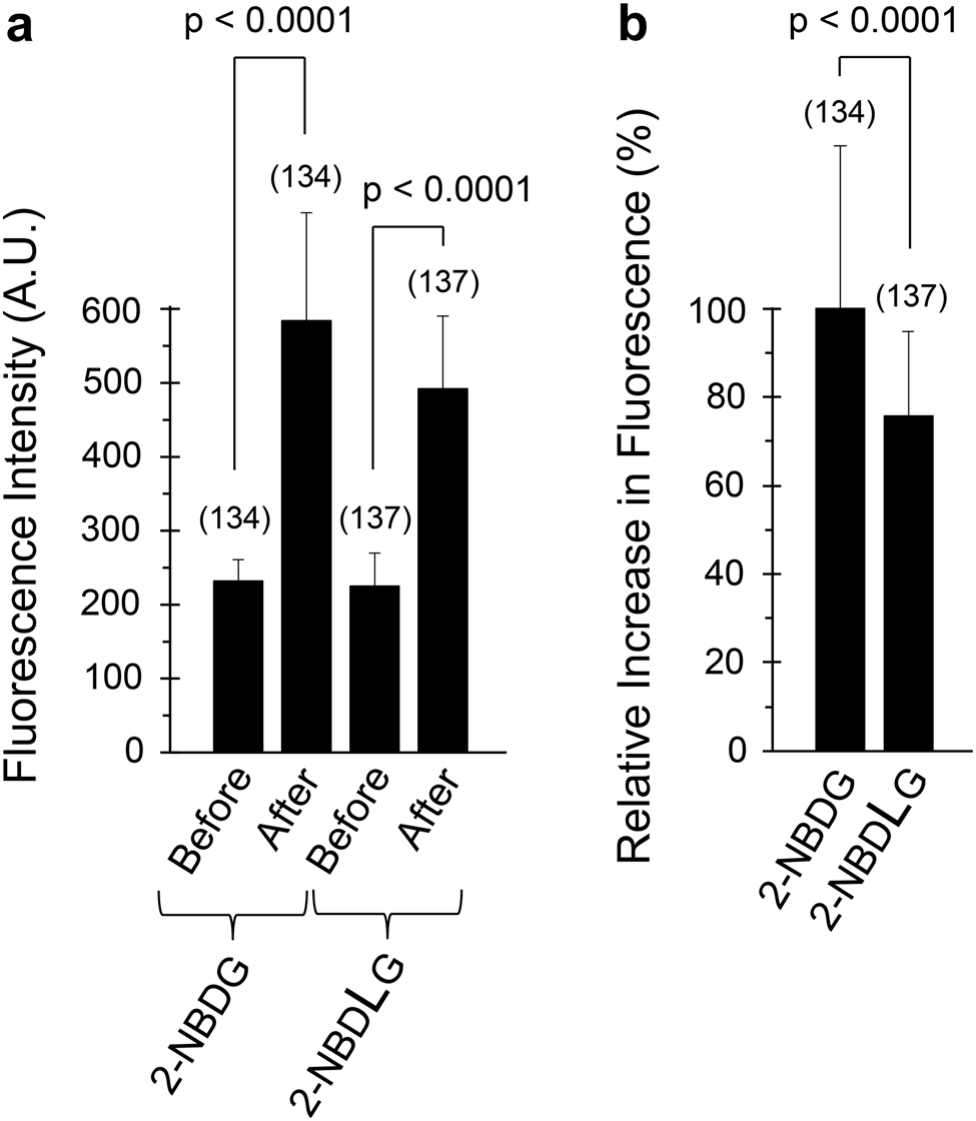
Quantitative evaluation of the 2-NBDG and 2-NBDLG uptake into U2OS cells examined by a laser confocal microscope. **a** Changes in the mean fluorescence intensities of U2OS cells examined at 7 DIV before and after administration of KRB solution containing 200 μM of 2-NBDG or 2-NBDLG. Data are the means ± SD of total fluorescence intensity of individual ROIs. **b** Net increase in the fluorescence in (a) was expressed as percent increase in the fluorescence relative to that for 2-NBDG administration. Numbers in parenthesis show the number of ROIs that were assigned as much as possible (14–24) for each of 7 pre-determined areas of the cover slip. Similar results were obtained in three separate experiments

U2OS cells took up not only a d-glucose analogue 2-NBDG, but also an l-glucose analogue 2-NBDLG (Fig. 3). A similar transient administration of KRB solution containing a mixture of 200 μM 2-NBDLG together with 20 μM 2-TRLG for 5 min produced a marked increase in the intensity of the fluorescence of U2OS cells in the green channel, although it appears less prominent to the case of the administration of 2-NBDG/2-TRLG mixture was administered (Fig. 3a–d). When averaged for seven areas, the fluorescence intensity of the U2OS cells in the green channel increased significantly after administration of the 2-NBDLG/2-TRLG mixture (491.9 ± 98.4 AU) from the value detected before administration (224.8 ± 45.1 AU, with the number of ROIs = 137, *p* < 0.0001, Fig. 4a).

In all areas tested for the administration of 2-NBDLG, U2OS cells consistently showed an increase in the fluorescence intensity in the green channel, although it was smaller than that for the administration of 2-NBDG (75.8 ± 19.0%, *p* < 0.0001, Fig. 4b). We have previously demonstrated a strong nuclear heterogeneity in mouse insulinoma MIN6 cells taking up 2-NBDLG [18]. Hence, we conducted nuclear staining of the U2OS cells immediately after finishing measurements of the fluorescence intensity on the stage of the confocal microscope. As demonstrated by DAPI staining, diverse nuclear shapes as well as sizes were found in U2OS cells tested (Online Resource 1).

A similar uptake of 2-NBDLG was consistently detected for U2OS cells in experiments performed in triplicate, although the net increase in the fluorescence intensity for administration of 2-NBDLG was always smaller than that of 2-NBDG (81.1 ± 9.8%, *n* = 3, *p* < 0.05). In other words, the difference indicates that a stereoselective mechanism participates in the uptake of 2-NBDG at least partly as we reported for MIN6 cells [18].

### Effects of antagonist of glucose transporters on the uptake of 2-NBDG/2-NBDLG in U2OS cells

**Fig. 5:**
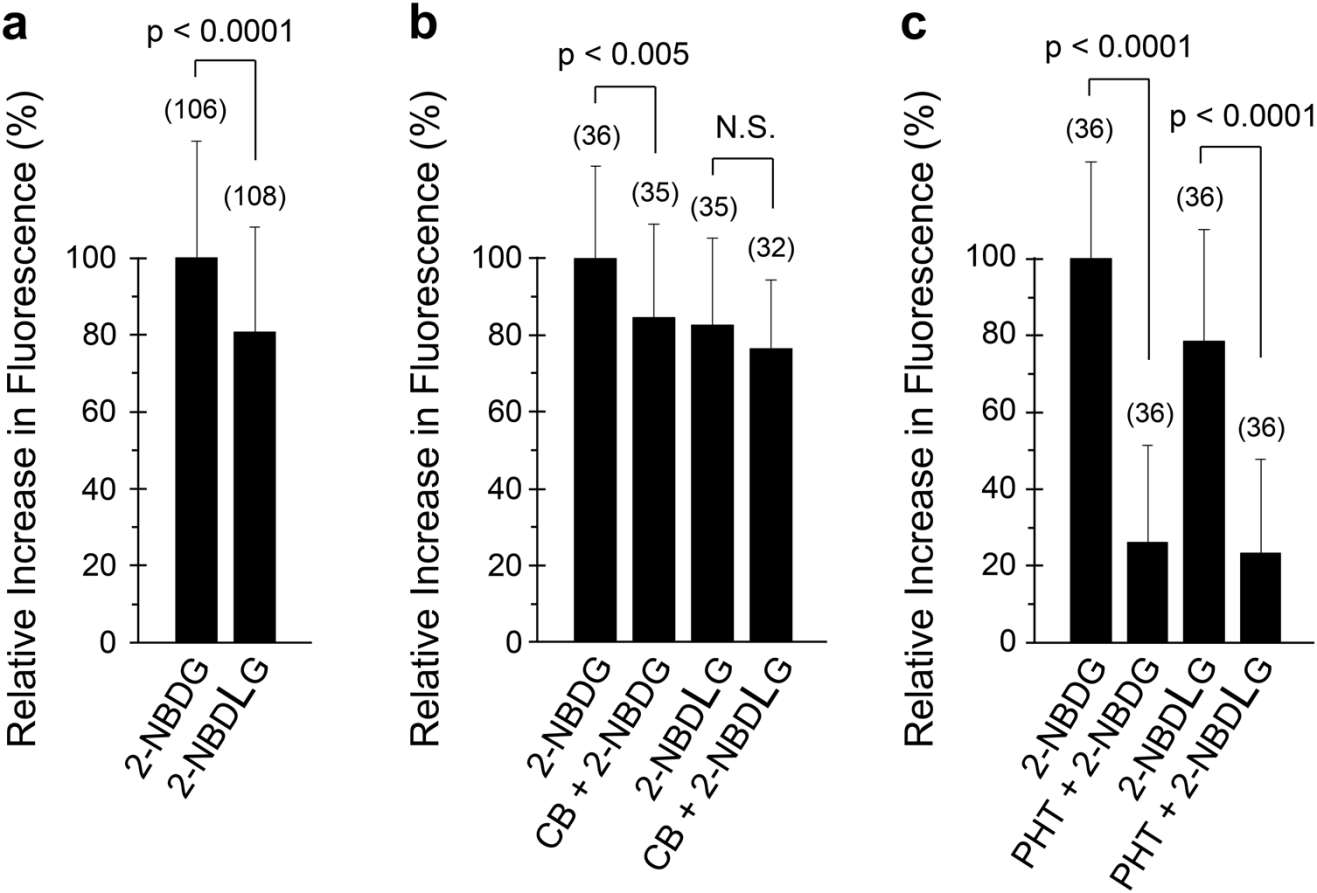
Pharmacological characteristics of 2-NBDG and 2-NBDLG uptake in U2OS cells evaluated by a fluorescent microplate reader. **a** Relative increase in the fluorescence intensity of U2OS cells for administration of 200 μM of 2-NBDG or 2-NBDLG examined at 7 DIV. Similar results were obtained in three separate experiments. **b** Effect of a GLUT inhibitor cytochalasin B (10 μM, CB) on the uptake of 2-NBDG and 2-NBDLG examined at 8 DIV. Similar results were obtained in two independent experiments. **c** Effect of phloretin (150 μM, PHT) on the uptake of 2-NBDG and 2-NBDLG examined at 7 DIV. Results were confirmed in experiments done in triplicate. Data are the means ± SD of percent increase in the fluorescence relative to the fluorescence increase for 2-NBDG administration on the same 96-well plate. Numbers in parenthesis show the number of ROIs that were assigned for each well of the same condition in the same 96-well plate as described in the Methods

It is difficult to compare the uptake of 2-NBDLG in U2OS cells to that of 2-NBDG simultaneously by confocal microscopy, since these tracers emit identical green fluorescence. Use of a fluorescence microplate reader enables such a comparison with a large number of cells simultaneously in a variety of pharmacological conditions [18]. When the average fluorescence of U2OS cells was evaluated in 96-well plates, remarkable increases in the fluorescence intensity was detected by administration of not only 2-NBDG, but also 2-NBDLG (Fig. 5a), in a very similar manner to those detected by confocal microscopy (Fig. 4b). The ratio of the net increase in the green fluorescence of U2OS cells for administration of 2-NBDLG to that of 2-NBDG was 80.9 ± 27.2%; the difference was significant between the uptake of these tracers (*p* < 0.0001, Fig. 5a). The average ratio of the net increase in the fluorescence for administration of 2-NBDLG to that of 2-NBDG was 78.7 ± 2.0% in triplicated experiments; the latter was consistently larger than that of the former, indicating a stereoselective uptake of the fluorescent d-glucose tracer in U2OS cells (*p* < 0.0001).

To pharmacologically characterize the uptake of these fluorescent glucose tracers in U2OS cells, we examined effects of specific antagonists of glucose transporters on the uptake (Fig. 5b). In the presence of 10 μM cytochalasin B, a potent antagonist of GLUTs, the uptake of 2-NBDG into U2OS cells was decreased significantly to 84.6 ± 24.2% (*p* < 0.005) of the control value obtained in the absence of cytochalasin B (100.0 ± 24.1%) (Fig. 5b). In contrast, cytochalasin B failed to significantly reduce the uptake of 2-NBDLG, while a large portion of the fluorescence remained intact (the relative ratio of 2-NBDLG uptake in the presence to the absence of cytochalasin B was 92.8 ± 21.5%; *p* = 0.280, Fig. 5b). Similar results were reproduced in an independent experiment, wherein the ratio of the fluorescence intensity was decreased significantly in the presence of cytochalasin B for administration of 2-NBDG (*p* < 0.0001, *n* = 32–36), but not for that of 2-NBDLG (*p* = 0.3621, *n* = 34–36). In duplicated experiments, the average ratio of the fluorescence intensity in the presence to the absence of cytochalasin B was 99.2 ± 9.0% for the administration of 2-NBDLG, whereas it was 77.5 ± 10.1% for the administration of 2-NBDG in average. These results suggest that GLUTs participate in the uptake of 2-NBDG, at least partly, but not in that of 2-NBDLG in U2OS cells.

A contribution of SGLTs, energy-demanding Na^+^-coupled glucose transporters, in the uptake of these tracers was unlikely, because neither uptake of 2-NBDG (*p* = 0.231, *n* = 72) nor 2-NBDLG (*p* = 0.444, *n* = 70–72) was significantly affected by 150 μM of phlorizin, a potent antagonist of SGLTs (Online Resource 2). A similar result was obtained in an independent experiment, wherein neither uptake of 2-NBDG (*p* = 0.933, *n* = 72) nor 2-NBDLG (*p* = 0.755, *n* = 72) was significantly attenuated by the same amount of phlorizin.

### A possible involvement of a non-stereoselective, non-GLUT/non-SGLT-mediated transport in the uptake of 2-NBDLG in U2OS cells

Persistence of the 2-NBDLG uptake in the presence of both cytochalasin B and phlorizin suggested an involvement of a non-GLUT, non-SGLT-mediated mechanism in the uptake of 2-NBDLG in U2OS cells. It is widely accepted that glucose transporters, at least those known to date, all operate by binding of d-glucose to their glucose binding site [4]. Alternatively saying, the uptake of fluorescently labeled glucose analogues could well be competitively inhibited by d-glucose, if it is mediated by glucose transporters. Thus, we examined an effect of a large amount of d-glucose or l-glucose on the uptake of 2-NBDLG.

In U2OS cells, however, 50 mM of d-glucose failed to inhibit 2-NBDLG uptake (*p* = 0.509, *n* = 17–27), whereas a slight, but significant, inhibition was detected for the uptake of 2-NBDG with the same amount of d-glucose (*p* = 0.011, *n* = 18–27; Online Resource 3). 50 mM l-glucose also failed to inhibit 2-NBDLG uptake into U2OS cells (*p* = 0.072, *n* = 18–27). A moderate inhibition (88.8 ± 0.9% of control in duplicated experiments in average) of the 2-NBDG uptake with 50 mM d-glucose (Online Resource 3) appeared consistent to the relatively small attenuation of 2-NBDG uptake by cytochalasin B (Fig. 5b). Taken together, these results support the idea that a non-stereoselective, possibly non-transporter-mediated mechanism underlies in the uptake of 2-NBDG as well as 2-NBDLG in U2OS cells.

In MIN6 cells, we have demonstrated that phloretin, a broad-spectrum inhibitor of channels and transporters including water channels and GLUTs [19, 20], abolished the uptake of 2-NBDLG as well as 2-NBDG [18]. Intriguingly, a striking reduction was detected in both uptake of 2-NBDLG and 2-NBDG into U2OS cells by 150 μM of phloretin, while a similar, small amount of fluorescence remained (*p* < 0.0001, Fig. 5c). The relative ratio of the uptake of 2-NBDLG and 2-NBDG in the presence of phloretin to that of 2-NBDG in the absence of phloretin was 23.4 ± 24.3% (*p* < 0.0001, *n* = 36) and 26.1 ± 25.3% (*p* < 0.0001, *n* = 36), respectively. No significant difference was detected between the relative fluorescence of 2-NBDLG and 2-NBDG in the presence of phloretin (Fig. 5c, *p* = 0.662, *n* = 36), implying a common, non-stereoselective component in the uptake of the fluorescent analogue of L- and d-glucose in U2OS cells. In triplicated experiments, the relative increase in the fluorescence intensity of U2OS cells in the presence to the absence of phloretin was 28.9 ± 1.1% for 2-NBDLG (*p* < 0.0001, *n* = 3) and 26.0 ± 0.4% for 2-NBDG (*p* < 0.0001, *n* = 3) administration in average. No significant difference was detected between the residual components of 2-NBDLG and 2-NBDG uptake in the presence of phloretin in triplicated experiments (*p* = 0.3014).

In summary, both pharmacological characterizations identified by a fluorescent microplate reader and the changes in the fluorescence intensity imaged by confocal microscopy have demonstrated that U2OS human osteosarcoma cells take up fluorescently labeled glucose analogues via non-stereoselective and stereoselective pathways. Especially, the uptake of 2-NBDLG, and of a large portion of 2-NBDG, occurred in a phloretin-dependent manner through a non-stereoselective, possibly non-transporter-mediated pathway, whereas a small portion of 2-NBDG uptake occurred through a stereoselective, most likely GLUT-mediated mechanism that was inhibited by both cytochalasin B and d-glucose.

## Discussion

In the present study, we demonstrated that human osteosarcoma U2OS cells took in a fluorescent analogue of l-glucose 2-NBDLG abundantly as well as its mirror image isomer 2-NBDG, a widely used fluorescent analogue of d-glucose. The ratio of the uptake of 2-NBDLG to that of 2-NBDG was very similar in confocal microscopy and fluorescent microplate reader experiments, 81.1 ± 9.8% and 78.7 ± 2.0%, respectively, in average. Pharmacological experiments further revealed that the uptake of 2-NBDLG in U2OS cells was markedly inhibited by phloretin, and not by a potent GLUT inhibitor cytochalasin B (Fig. 5). This contrasted the weak but significant attenuation by cytochalasin B for the uptake of 2-NBDG in U2OS cells, while a large portion of the uptake persisted in the presence of cytochalasin B (Fig. 5b).

Interestingly, phloretin remarkably reduced the uptake of not only 2-NBDLG but also 2-NBDG in U2OS cells leaving only a small fluorescence component at a comparable intensity (Fig. 5c). In addition, no significant effect was detected of phlorizin, a potent inhibitor of SGLTs, on either 2-NBDLG or 2-NBDG uptake (Online Resource 2). These characteristics of the uptake in U2OS cells are very similar to those we reported previously in MIN6 cells [18], suggesting a non-GLUT/non-SGLT, yet unidentified mechanism participates in the uptake of the glucose analogues in the tumor cells derived from different organs of different species.

It has been postulated that cellular uptake of d-glucose by glucose transporters including GLUTs and SGLTs is initiated by binding of d-glucose to the glucose-binding site in the transporters, producing changes in their conformation from outward-facing to inward-facing, enabling a movement of d-glucose across the plasma membrane to the intracellular space [29]. The point is that the binding of d-glucose is a necessary condition for the uptake by glucose transporters to occur. In addition, it has been well established that 2-NBDG is taken up into mammalian cells through glucose transporters such as GLUTs and SGLTs [4]. Consistently, previous studies, including those by our group, have demonstrated that the uptake of 2-NBDG in mammalian cells is competitively inhibited by d-glucose [4, 11, 30].

In contrast, in the uptake of 2-NBDLG in U2OS cells, no significant inhibition was detected by a large amount (50 mM) of d- nor l-glucose, although 2-NBDG uptake into U2OS cells was attenuated significantly by d-glucose (Online Resource 3). Results obtained by these functional studies appear consistent to an idea that U2OS cells express at least two pathways for taking up the fluorescent analogues of glucose; a phloretin-inhibitable, possibly non-transporter-mediated pathway that enables the uptake of both fluorescently labeled l- and d-glucose analogues in a non-stereoselective manner, and a conventional glucose transporter such as a GLUT-mediated pathway that carries the D-form predominantly [4].

Consistent to the above notion, previous studies have shown that osteosarcoma cells including U2OS cells express GLUTs [31–34]. 2-NBDG uptake has also been reported in some osteosarcoma cells [33, 35]. To our best knowledge, however, the stereo-preference in the uptake of fluorescently labeled glucose has never been examined in osteosarcoma cells, while there is a study that reported an ignorable uptake of [^3^H]-l-glucose and much larger uptake of [^3^H]-d-glucose in cultured chicken osteoclasts isolated from medullary bone [36].

Although U2OS cells evaluated in the present study exhibited a stereo-preference for the uptake of fluorescently labeled d-glucose over l-glucose (Fig. 4b, 5a) as we previously reported in MIN6 cells [18], the rate of the uptake of the l-glucose analogue to that of the d-glucose analogue was much larger in U2OS cells (approximately 80%) than that in MIN6 cells (approximately 40–50%). It is unclear why the stereo-preference in the uptake differs in these two tumor cells. Osteosarcoma is often comprised of pathologically heterogeneous populations of cells [23]. Indeed, the extent of the uptake of the fluorescent d- as well as l-glucose analogue was inhomogeneous among U2OS cells tested (Fig. 2d, 3d). Considerable heterogeneity was shown in cellular and nuclear shapes of U2OS cells as well (Online Resource 1). We, therefore, speculate that there is a difference between these two tumor cells in the percentage of a sub-population of cells capable of taking up more 2-NBDLG than others. Consistent to this hypothesis, we have reported that a relatively small portion of MIN6 cells expressing malignant phenotypes exhibited a remarkable uptake of 2-NBDLG, whereas only minimum uptake could be detected in other MIN6 cells showing no such phenotypes [18]. It is of interest to isolate such cells and to identify molecular mechanisms responsible for the uptake of l-glucose analogues. Phloretin blocks a wide variety of membrane transports that are mediated by not only transporters but also channels like water channels, and that some water channels transport carbohydrates such as glycerol [37]. A strong demand for nutrients by proliferating tumor cells might work in favor of cells expressing channel-like versatile mechanism with a lowered specificity for ligands [4]. Fluorescence lifetime imaging (FLIM) and fluorescence resonance energy transfer (FRET), techniques for imaging inter-molecular interactions, may provide further validation, when molecules possibly responsible for the uptake are proposed [38, 39].

### Future perspectives

d-glucose is one of the most fundamental nutrients for living things. To visualize this glucose uptake at the single cell resolution, 2-NBDG has been effectively used in both normal cells and tumor cells isolated from various organs including those from patients [40–42]. One drawback is that 2-NBDG cannot discriminate tumors from non-tumor tissues, especially fat, muscle, or inflammatory lesions similarly to with FDG-PET imaging [5, 7, 43, 44].

Use of l-glucose analogues might help overcome this problem. An l-glucose tracer 2-NBDLG is taken up predominantly in tumor cells expressing malignant phenotypes as we have reported in in vitro by MIN6 mouse pancreatic tumor cells [18] and in vivo by hamster bile duct cancer [45]. In the present study, we further demonstrated that human osteosarcoma U2OS cells take in 2-NBDLG showing pharmacological properties that are very similar to those in MIN6 cells. Moreover, a combinatory use of 2-NBDLG with a membrane-impermeable 2-TRLG enables identification of the tumor excluding non-specific entry of the tracers in inflammatory and/or damaged lesions [4, 18, 45].

There is a long-standing controversy over whether the high amount of glucose uptake in malignant tumor cells in FDG-PET imaging can solely be explained by an overexpression of conventional glucose transporters such as GLUTs and/or SGLTs [4]. Less is known about non-transporter-mediated uptake of glucose in both physiological and pathophysiological conditions [4, 37, 46]. Further studies on the aberrant uptake of fluorescent L-glucose analogues might provide clues to explore the mechanism of nutrient uptake commonly used by these aggressive neoplasms; osteosarcoma and bile duct/pancreatic cancers.

## Supplementary Information

Below is the link to the electronic supplementary material.

Supplementary file1 (PDF 196 KB)

Supplementary file2 (PDF 32 KB)

Supplementary file3 (PDF 84 KB)

Supplementary file4 (DOCX 27 KB)
