# Supplementary material for: Uptake of fluorescent d- and l-glucose analogues, 2-NBDG and 2-NBDLG, into human osteosarcoma U2OS cells in a phloretin-inhibitable manner
Source: Hum Cell. 2021 Jan 17;34(2):634–43. doi: 10.1007/s13577-020-00483-y (PMC7900340; doi:10.1007/s13577-020-00483-y)
Supplement: Supplementary file 1 — Supplementary file1 (PDF 196 KB) [file 13577_2020_483_MOESM1_ESM.pdf]

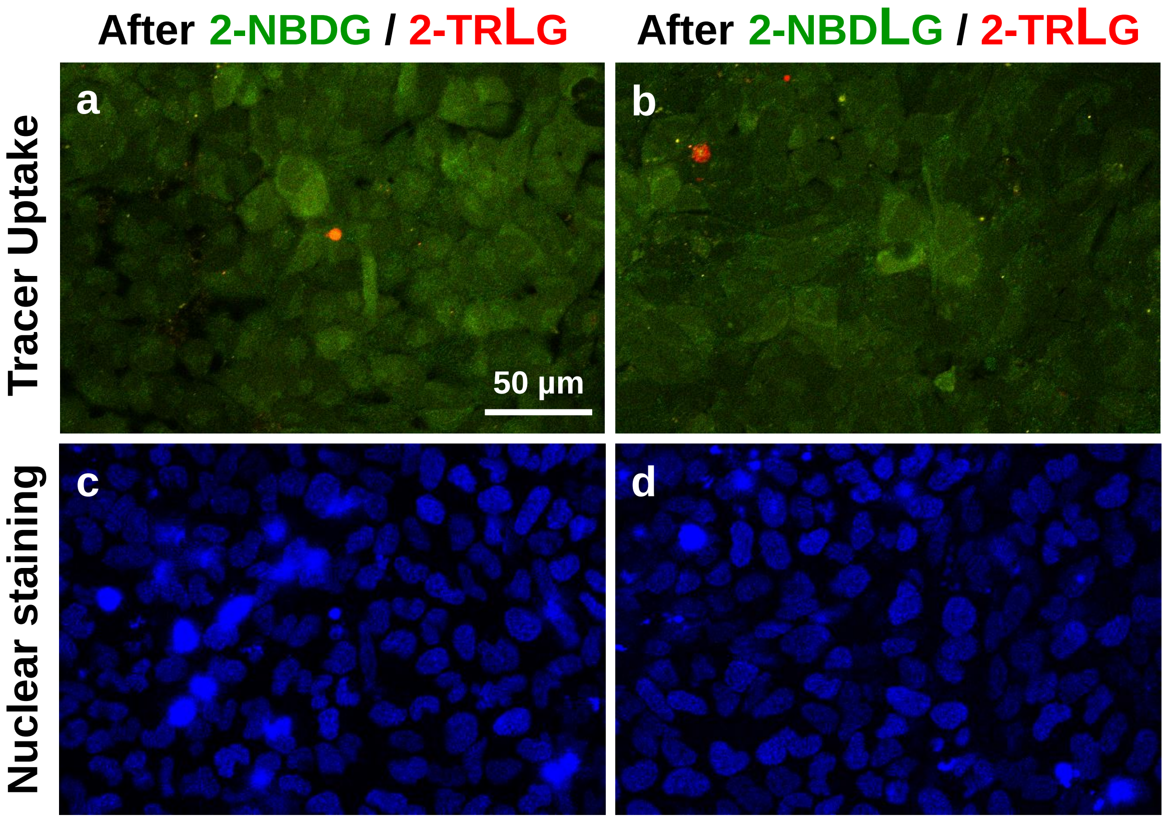

Online Resource 1. Representative live nuclear imaging of U2OS cells with DAPI conducted immediately after the measurement of 2-NBDG and 2-TRLG (a and c), or of 2-NBDLG and 2-TRLG (b and d), uptake on the stage of a confocal microscope. **a,** A superimposed image of fluorescence of U2OS cells captured in 500-580 nm (green channel) and in 580-740 nm (red channel) after administration of 200 µM 2-NBDG and 20 µM 2-TRLG for 5 minutes. The image was reproduced from Fig. 2h. **b,** Similar to (a), but after administration of 200 µM 2-NBDLG and 20 µM 2-TRLG. **c and d,** Nuclear staining of cells in (a) and (b) with DAPI, respectively. Note that cells as well as nuclei of variable size and shape, such as large, small or elongated, are seen. The bar is common to all panels. We have shown that living tumor cells expressing malignant phenotypes are stained with DAPI, although the fluorescence intensity of nuclei greatly varies among cells [18].
