# Supplementary material for: Uptake of fluorescent d- and l-glucose analogues, 2-NBDG and 2-NBDLG, into human osteosarcoma U2OS cells in a phloretin-inhibitable manner
Source: Hum Cell. 2021 Jan 17;34(2):634–43. doi: 10.1007/s13577-020-00483-y (PMC7900340; doi:10.1007/s13577-020-00483-y)
Supplement: Supplementary file 2 — Supplementary file2 (PDF 32 KB) [file 13577_2020_483_MOESM2_ESM.pdf]

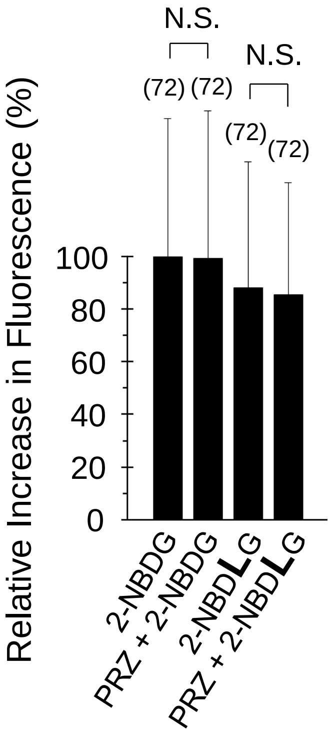

Online Resource 2. Effect of phlorizin (150 μM, PRZ), a potent inhibitor of SGLTs, on the uptake of 2-NBDG and 2-NBDLG in U2OS cells examined at 7 DIV with a fluorescent microplate reader. No significant decrease in the fluorescence of U2OS cells was produced by PRZ for the administration of 200 μM 2-NBDG as well as the same amount of 2-NBDLG. Values are expressed as the relative increase in the fluorescence of cells compared to the mean fluorescence increase detected for administration of 2-NBDG on the same 96-well plate. Numbers in parenthesis represent the number of ROIs measured. The same results were obtained in experiments performed in duplicate.
