# Supplementary material for: Uptake of fluorescent d- and l-glucose analogues, 2-NBDG and 2-NBDLG, into human osteosarcoma U2OS cells in a phloretin-inhibitable manner
Source: Hum Cell. 2021 Jan 17;34(2):634–43. doi: 10.1007/s13577-020-00483-y (PMC7900340; doi:10.1007/s13577-020-00483-y)
Supplement: Supplementary file 3 — Supplementary file3 (PDF 84 KB) [file 13577_2020_483_MOESM3_ESM.pdf]

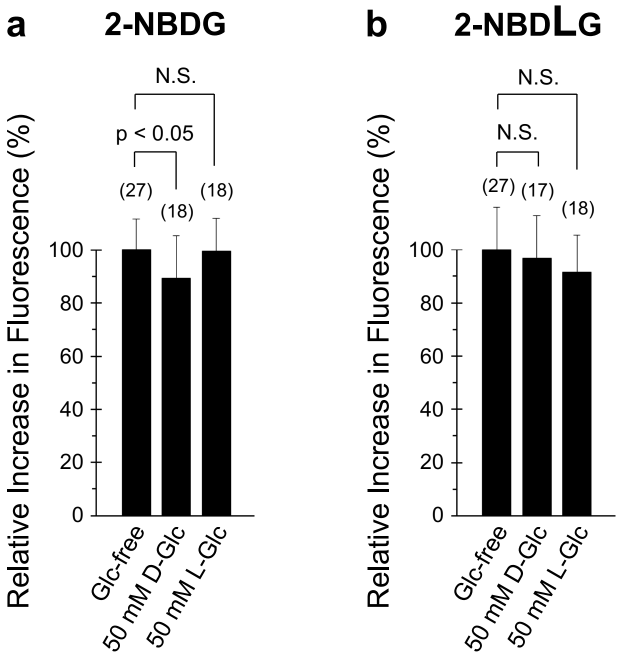

Online Resource 3. Effect of a large amount of D- or L-glucose on the uptake of 2-NBDG (a) and 2-NBDLG (b) in U2OS cells examined at 8 DIV with a fluorescent microplate reader. **a,** A slight, but significant ($p < 0.05$), attenuation by 50 mM D-, but not L-glucose, was detected in the fluorescence intensity of cells for administration of 200 μM of 2-NBDG, whereas no significant reduction in the fluorescence was detected by the same amount of L-glucose. **b,** Similar to (a), but for the uptake of 2-NBDLG. Neither a large amount of D-glucose nor L-glucose could significantly reduce the 2-NBDLG uptake into U2OS cells. Numbers in parenthesis represent the number of ROIs measured. The same results were obtained in experiments performed in duplicate.
