# Supplementary material for: Uptake of fluorescent d- and l-glucose analogues, 2-NBDG and 2-NBDLG, into human osteosarcoma U2OS cells in a phloretin-inhibitable manner
Source: Hum Cell. 2021 Jan 17;34(2):634–43. doi: 10.1007/s13577-020-00483-y (PMC7900340; doi:10.1007/s13577-020-00483-y)
Supplement: Supplementary file 4 — Supplementary file4 (DOCX 27 KB) [file 13577_2020_483_MOESM4_ESM.docx]

**ONLINE RESOURCE**

**Online Resource 4. Methods in detail.**

**Confocal microscopic imaging**

***Culture***

Osteosarcoma U2OS (HTB-96, ATCC) cells were cultured using RPMI 1640 medium (11875-093, Gibco) containing 10% Fetal Bovine Serum (26140-079, Gibco) and 1% Penicillin - Streptomycin (15140-122, Gibco). Cells in passages from 32 - 36th were used in the experiment. 10 μl of U2OS cell suspension (5000 cells/ml) was seeded on small glass coverslips (2.5 mm times 7 .0 mm, No. 0, Matsunami Glass Ind., Ltd.). After leaving cells for 60 minutes in a CO_2_ incubator at 37ºC for ensuring stable attachment, 500 μl of the culture medium was carefully added to each well. Culture medium was half exchanged every 3 days.

***Measurement***

Confocal microscopy was conducted to visualize difference in the uptake of the fluorescent D- and L-glucose tracers in U2OS cells. Cells used for the measurement were cultured for 7 days *in vitro* (DIV) when an adequate number of cells maintaining healthy condition were obtained for each coverslip at the concentration seeded. The tracer administration and image acquisition were conducted by modifying a method reported previously [7, 27]. In brief, the glass coverslip was placed on a temperature-controlled, custom-made chamber, which was held on a motor-driven xyz stage of a laser confocal microscope (TCS-SP5, Leica). Cells were continuously superfused with Krebs Ringer Buffer Solution (KRB) (in mM**;** 129 NaCl, 4.8 KCl, 1.2 KH_2_PO_4_, 1.2 MgSO_4_, 1.0 CaCl_2,_ 10 HEPES, 5.0 NaHCO_3_, 0.5 D-glucose, 0.1 carbenoxolone, pH 7.30 - 7.35) at 37ºC at a rate of 1.0 ml per minute.

The fluorescent tracer was administrated for 5 minutes by transiently exchanging the KRB solution with that containing 200 μM of 2-NBDG and 20 μM of 2-TRLG (Fig 1c), a membrane-impermeable, red fluorescence-emitting L-glucose analogue that bears a large, charged fluorophore Texas Red [27]. Immediately after finishing the tracer uptake measurement, cells were incubated with DAPI for 30 minutes at 37ºC on the confocal microscope stage for nuclear staining. The nucleus was visualized in the wavelength range 415-478 nm using a 405 nm laser source [18].

The tracers were excited by a single 488 nm laser source. The fluorescence of cells was simultaneously detected in the wavelength range 500-580 nm (hereinafter, green channel) and 580-740 nm (red channel) for evaluating 2-NBDG/2-NBDLG and 2-TRLG fluorescence, respectively, with a dichroic mirror at 500 nm (RSP 500) and a spectroscopy method using prisms and slits of TCS-SP5. In this acquisition setup, the green channel contains fluorescence originated from 2-NBDG/2-NBDLG with only negligible fluorescence from 2-TRLG, while the red channel contains a strong fluorescence of 2-TRLG and a weak one of 2-NBDG/2-NBDLG that have a small component in the wavelength longer than 580 nm [27]. The objective lens used was HCX PL APO 40x/1.25-0.75 OIL.

To evaluate the uptake of the tracers in U2OS cells, seven areas were selected for each of the coverslips, then round-shaped regions of interest (ROIs; diameter = 65 μm, n = 14-24 for each area) were assigned using LAS X software (Leica). The total fluorescence intensity of individual ROIs was averaged and compared for all of the ROIs, areas, and coverslips, before and 6 minutes after administration of either a mixture of 2-NBDG and 2-TRLG, or of 2-NBDLG and 2-TRLG. Regions containing apparent debris or cells only sparsely were excluded from the ROI assignment. ROIs showing an apparent 2-TRLG permeation were also omitted from the ROI assignment, because in such ROIs non-specific entry of 2-NBDG/2-NBDLG might occur due to a loss of membrane integrity [18].

**Pharmacological evaluation of the tracer uptake by a fluorescence microplate reader**

***Culture***

Details in the culturing and experimental procedures were similar to those reported previously [18]. In brief, 10 μl cell suspension at a concentration of 1 x 10^6^ cells/ml was plated on the center of each well from column 2 to column 9 of a 96-well plate (μClear-plate #655090, Greiner Bio-One) except for the top (A) or the bottom (H) rows wherein no cell was contained in an alternating manner (see also Measurement). After 30 minutes’ incubation at 37ºC in a CO_2_ incubator, 200 μl of the culture medium was further added to each well, resulting in a density of 1 x 10^4^ cells/well. Culture medium was half-exchanged every 3 days. Cells at 7 or 8 DIV were used for measurement.

***Measurement***

Just before measurements, culture medium was removed from each well leaving 50 μl. Cells were then washed four times with 350 μl of standard Krebs Ringer Buffer Solution (KRB) (in mM; 129 NaCl, 4.8 KCl, 1.2 KH_2_PO_4_, 1.2 MgSO_4_, 1.0 CaCl_2,_ 10 HEPES, 5.0 NaHCO_3_, 0.5 D-glucose, 0.1 carbenoxolone, pH 7.30 - 7.35) at room temperature (26 ± 1ºC) by a microplate washer (AquaMax, Molecular Devices). After the fourth wash, KRB was added to adjust the height of solution to that of blank wells in column 10, in which 200 μl of KRB not containing tracers was added.

For the measurement, a fluorescence microplate reader was used with its operation software (FlexStation and SoftMax Pro, Molecular Devices). The fluorescence intensity was measured from the bottom of the plate three times and was averaged. Excitation, emission, and cut off wavelength were 470 nm, 540 nm, and 495 nm, respectively. Nine ROIs, 1.5 mm in diameter, were assigned for individual wells of the 96-well microplate. All wells were scanned before and after the experiment with a flatbed scanner (GT-X820, Seiko Epson). ROIs, in which cells were unevenly arranged or lost during the washout procedure, were excluded from the analysis.

The microplate reader experiments were conducted according to a precisely timed protocol. Before starting experiments, the autofluorescence of cells was measured for individual ROIs. Using an 8 channel pipette, 50 μl of KRB solutions containing either 400 μM of 2-NBDG, or the same amount of 2-NBDLG, were simultaneously added into the 7 wells, in which 50 μl of KRB was pre-loaded (the final concentration of the tracer was 200 μM), from rows A to G in columns 2-9 (B to H in some columns) in an alternating manner. Wells to which no tracer was administered were used to check changes in the autofluorescence during the experiment. The alternating arrangement of the tracers assured reliable evaluation of the fluorescence eliminating inhomogeneity in the system including subtle variability of washout process among wells. Wells in the top (A) rows for the former, and the bottom (H) for the latter, were used as control wells to examine if the tracers were successfully washed out.

10 minutes later, 50 μl of the tracer solution was removed from the wells, then 200 μl of KRB was added manually according to the time-matched protocol. 350 μl of KRB solution was then added and removed 8 times at room temperature using the microplate washer except for the last wash, wherein 200 μl of KRB was left for the fluorescence measurement. Administration of KRB containing phloretin (150 μM) or phlorizin (150 μM) were performed similarly, except that the KRB solution was applied 5 minutes prior to the tracer administration.

In experiments investigating the effect of cytochalasin B on the uptake, cells were incubated in KRB solution containing no D-glucose for 40 minutes on a gently rotating shaker at 37ºC for depleting intracellular D-glucose storage. After the incubation, cells were incubated for another 5 minutes with KRB solution containing 10 μM of cytochalasin B prior to the tracer administration. Finally, KRB solution containing both the tracer and cytochalasin B was administered for 15 minutes. The osmolarity of KRB solution in the cytochalasin B experiment was measured (VAPRO Model 5520, Wescor) and adjusted by NaCl so that it was not changed between the control and experimental group. The effect of competitive inhibition by a large amount (50 mM) of D- or L-glucose on the uptake of 2-NBDLG and 2-NBDG was examined by slightly modifying the method described previously [18].
